# Baggio–Yoshinari Syndrome: A Report of Five Cases

**DOI:** 10.3390/microorganisms12102108

**Published:** 2024-10-21

**Authors:** Virginia Lucia Nazario Bonoldi, Natalino Hajime Yoshinari, Giusto Trevisan, Serena Bonin

**Affiliations:** 1 Reumatologia, Hospital das Clinicas HCFMUSP, Faculdade de Medicina, Universidade de São Paulo, São Paulo 05508-220, Brazil; virginia.bonoldi@hc.fm.usp.br (V.L.N.B.);; 2 Department of Medical Sciences, University of Trieste, 34149 Trieste, Italy; trevisan@units.it

**Keywords:** Baggio–Yoshinari syndrome, Borrelia, Lyme, ticks

## Abstract

Compared to classic Lyme disease (LD), Baggio–Yoshinari syndrome (BYS) has the following distinctive characteristics: it is transmitted in the Amazon area and Northeast, Central-West, Southeast, and South regions of Brazil by hard ticks, notably *Amblyomma cajannense* or *Rhipicefalus* sp. The absence of *Ixodes* sp. ticks in areas at risk of BYS in Brazil is probably the main reason for the disease’s differences from LD in the United States, Europe, and Asia. Biodiversity and climate probably favor the formation of atypical pleomorphic Borrelias, which have not yet been cultivated or isolated. Clinically, the first manifestation of BYS is the erythema migrans as in the classic forms of Lyme disease, but BYS is distinguished from LD by its prolonged clinical evolution, with a high frequency of relapses and the appearance of autoimmune manifestations. Prevalent symptoms are headache and erythema nodosum. Five clinical cases of BYS in patients who contracted the disease in the Brazilian Amazon rainforest are described here. This syndrome should be considered among differential diagnoses in patients bitten by ticks in Brazil who present with erythema migrans and/or headache. It is important to pursue an early diagnosis because symptoms respond well to antibiotics in the early stages; if treatment is started late, a chronic course with articular and neurological sequelae can be detected.

## 1. Introduction

Baggio–Yoshinari Syndrome (BYS) is an emerging tick-borne infectious disease discovered in Brazil in 1993 by Natalino Hajime Yoshinari and Domingos Baggio [1,2]. The study of Lyme disease (LD) began in Brazil in 1989. Since then, several cases of human Borreliosis have been reported in Brazil, the first manifestation of which has often been an erythema migrans, as in classic LD. However, epidemiological, clinical, and laboratory differences were noticed between LD as described in the Northern Hemisphere and the Brazilian LD, which in 1993 was named Baggio–Yoshinari Syndrome (BYS) [1].

In 2010, the presence of *Borrelia burgdorferi* sensu lato in the patients’ skin lesions was confirmed by immunohistochemistry and focus floating microscopy [3]. Polymerase chain reaction (PCR) procedures supported the causative role of *Borrelia burgdorferi sensu stricto* spirochete in BYS, although the bacterium has not yet been isolated or cultured. In addition to erythema migrans, BYS can present with other skin manifestations, such as the erythema nodosum [4]. Ocular symptoms can be observed, especially in the early phase of the disease. Muscle and joint involvement, neurological disorders, and cardiac manifestations have also been reported in BYS [4,5].

The Brazilian Lyme disease-Like illness is similar to LD; however, Brazilian borreliosis differs from LD as observed in the Northern Hemisphere for several characteristics. In Brazil, this zoonosis is transmitted to humans through the bite of *Amblyomma* and *Rhipicephalus* genera ticks, which do not belong to the *Ixodes* genus, whose ticks are the usual vectors of Lyme disease Borreliae [6]. *Amblyomma cajennense* (which can also transmit the spotted fever) is present especially in the Amazon rainforest, which extends for about 65% of the territory in Brazil, where BYS has been reported. Nevertheless, BYS can also be present in Colombia, Peru, Venezuela, Ecuador, Bolivia, Guyana, Suriname, and French Guiana [7]. The probable reservoir of the BYS is the Capybara (*Hydrochoerus hydrochaeris*), but domestic animals can also play a role in the diffusion of these spirochaetes. The infection can indeed affect horses in Brazil, but there is little documented evidence of this [8].

The adaptation of *Borrelia burgdorferi* to Brazilian vectors and reservoirs probably originated from spirochetes with atypical morphologies (cysts or cell-wall-deficient bacteria) exhibiting genetic adjustments, such as gene suppression. These details could explain the protracted survival of these bacteria in hosts beyond the induction of a weak immune response and the emergence of reactive severe symptoms [2]. At present, the only available sequence from a patient with BYS (HM245929) refers to a fragment of 329 bp of the Flagellin E gene that, when compared with *B. burgdorferi* sensu stricto (*Bbss*), shows a difference in 2-8 substitutions. This is the only distinctive feature that can serve as a fingerprint for Brazilian Borrelia [9].

This report aims to highlight the differences between BYS and classical LD by describing five cases in which the diagnosis was made using the criteria developed by the University of São Paulo. This report would aid health professionals in recognizing this exotic and neglected zoonosis [10].

## 2. Patients and Methods

Patients described hereafter were diagnosed in Brazil and followed up at the Dermatology Unit of the University of Trieste from 2014 to 2017, in agreement with Prof. Yoshinari, to whom this article is dedicated. All patients contracted BYS in the Amazon, where they were diagnosed based on the criteria described hereafter. In Brazil, at the time of diagnosis, serological tests were carried out using an in-house method as already described [11]. Four patients came back to Italy where they were followed jointly with the Unit of Rheumatology of the Hospital das Clinicas. Patient number 5 was diagnosed and followed only in Brazil in the Hospital das Clinicas. In addition to a careful anamnesis in Italy, some patients were re-submitted to serological tests for the detection of Lyme Borreliosis and, in 2 cases, to PCR analyses for Borrelial DNA detection. PCR tests were carried out in Italy by using DNA obtained from blood and in one case from tear fluid and by amplifying a fragment of the Flagellin B gene of 98 bases, using the following primers Fw primer 5’-GGAGCAAACCCAAGATGAAGC-3’, reverse primer 5’-GGTGCAGCCTGAGCAGTT-3’. Amplicons were separated and analyzed by fragment analysis at the capillary electrophoresis.

The diagnosis of BYS was made following the diagnostic criteria for the Brazilian Lyme disease-like illness developed by the “Laboratório de Investigação Médica-17 (LIM-17), Hospital das Clínicas da Faculdade de Medicina da Universidade de São Paulo” as follows:

Major criteria

- Epidemiology (tick bite or contact with wild or domestic animals in risk areas).
- Erythema migrans or systemic manifestation (arthritis, neurological abnormalities, cardiac involvement).
- Positive serology for *Borrelia burgdorferi*.

Minor criteria

- Relapsing symptoms.
- Chronic fatigue or cognitive disturbances.
- Identification of spirochete-like structures by dark-field microscopy or Giemsa staining.

A positive diagnosis was made if the results for three major criteria or two major and two minor criteria were met [4,5].

## 3. Case Reports

The patients reported hereafter contracted BYS in the Amazon; four of them (cases 1–4) also lived or worked in Italy. Table 1 summarizes each patient’s symptoms and test results.

### 3.1. Case 1

**Figure 1:**
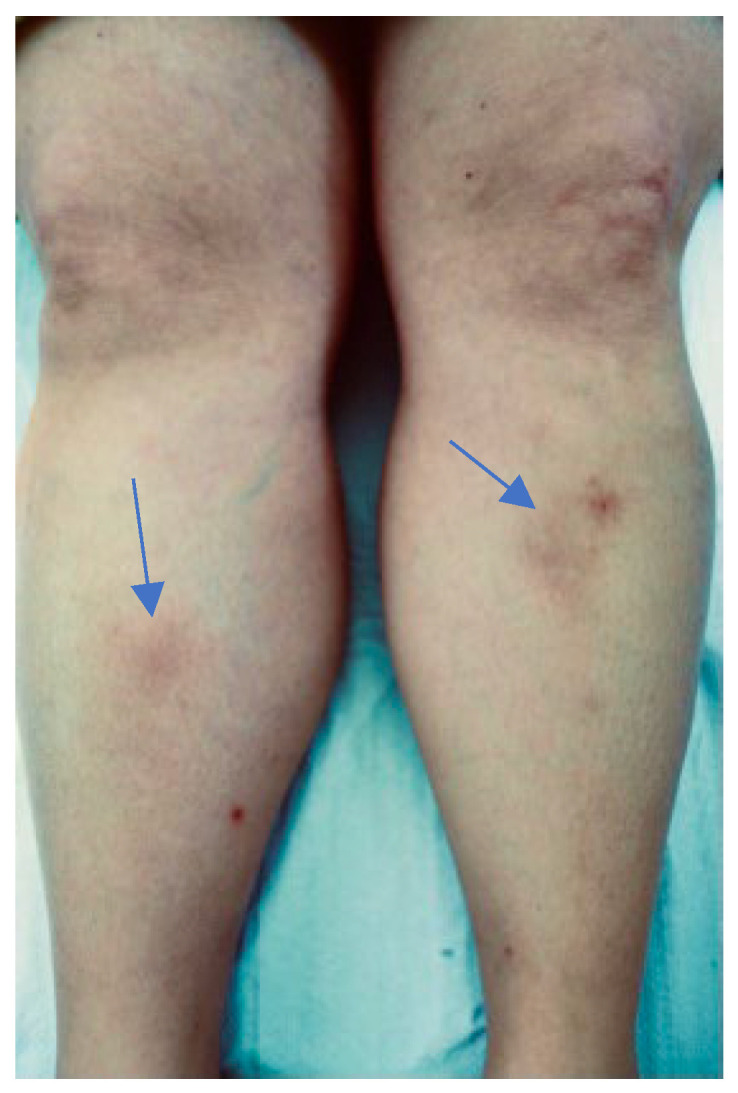
Erythema nodosum developed in patient 1 in 2014.

A 47-year-old woman who lived in Brazil for 5 years and frequented the Amazon reported a tick bite by *Amblyomma cajennense*, on her left leg when she was 45. After two weeks, she developed an erythema migrans around the tick bite. Subsequently, she also reported headache, as well as migrating arthralgia, reactive left lateral cervical, axillary, and inguinal lymph nodes (on ultrasound with a clearly visible hilum), and recurrent erythema nodosum (Figure 1). At that time, laboratory tests showed positive anti-Bbsl IgM antibodies by ELISA. In Brazil, a diagnosis of BYS was made based on the positivity of 3 major criteria for BYS diagnosis.

In 2014, when she presented in Italy, she was negative to serological tests for *Borrelia burgdorferi sensu lato* (*Bbls*). She had positive anti-Ehrlichia IgG antibodies, anti-nuclear antibodies (ANAs) = 1:320, and creatine kinase (CK) = 433. The liquor cerebri test, MRI of the head and spine, and SPECT (Single-Photon Emission Computed Tomography) were negative.

Taken the clinical history of BYS and the persistence of the symptoms, the patient was treated with intravenous ceftriaxone 2 g/day for 30 days, followed by doxycycline 100 mg twice/day for 3 months in association with hydroxychloroquine 200 mg twice/day for 6 months, according to the Yoshinari therapeutic protocol for this syndrome.

One year after the treatment, the symptoms improved, the erythema nodosum and the lymphadenopathy regressed, the headache and asthenia subsided, brain MRI, SPECT, and anti-Bbsl antibodies were negative, the ANAs were reduced by one dilution (1:160), and the CK was 74.

### 3.2. Case 2

A 46-year-old Argentine woman lived from the age of 40 to 45 in Brazil. Since the age of 41, after excursions in the Amazon rainforest and several tick bites, she reported neurological symptoms with recurrent headache without aura and resistance to different therapies. She also reported constant low-grade fever, temporomandibular arthralgia, cognitive disorders, recurrent tendinitis, asthenia, and neck pain. In the following years, a progressive motor disability appeared, requiring the use of a wheelchair, as well as paresthesia of the lips, face, and hands, insomnia, difficulty focusing, lateral cervical microlymphadenopathy. PCR for Borrelia detection was positive in DNA obtained from tears but not from peripheral blood. Anti-nuclear antibodies (ANAs) were negative and neuron-specific enolase (NSE) antibodies were positive. Before coming to Italy, she had not undergone any treatment for LD. However, she was submitted to amoxicillin treatment for several episodes of otitis, with a significant benefit to her clinical BYS manifestations.

In the following years, the neurological symptoms worsened with a persistent headache and cervical myelopathy with dysautonomia, motor impotence, paresthesia of the lips, face, and hands, and temporomandibular arthritis. The patient was treated according to the guidelines of the Yoshinari protocol (ceftriaxone 2 g/iv for 30 days and doxycycline 200 mg/day for 2 months + hydroxychloroquine 400 mg/day for 6 months), with an improvement in symptoms, in particular those of headache, the motor disability of temporomandibular arthritis, and the disappearance of low-grade fever.

### 3.3. Case 3

**Figure 2:**
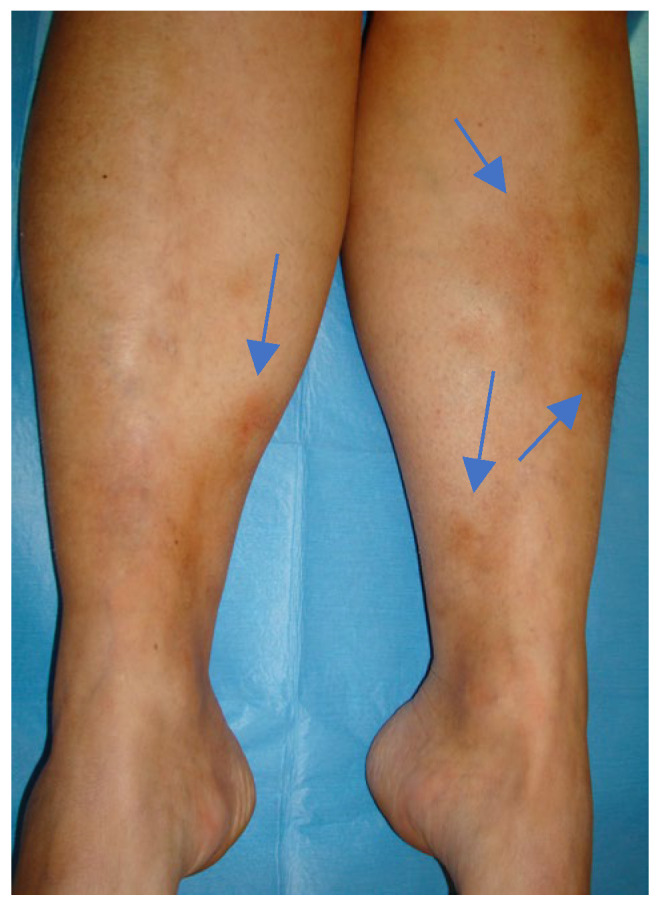
Erythema nodosum in patient 3 in 2017.

An Italian 35-year-old anthropologist who has often lived in Brazil for work since the age of 22 reported two tick bites on her right popliteal area and on her leg in 2009 in Minas Gerais, when she was 27. The tick was a *Amblyomma cajennense*. After a few weeks, she developed headaches, tinnitus, migrating arthralgia, and an erythema nodosum (Figure 2).

Anti-Bbsl IgM antibodies were positive by ELISA, PCR for Borrelia detection in blood was positive, while it was negative in the DNA obtained from the tear fluid. Autoantibodies (ANAs, ENAs) were negative. Considering the clinical manifestations and the history of the tick bites in Amazonia, two major and two minor criteria of BYS were met. The patient was treated with ceftriaxone 2 g/iv per day for a month and then with minocycline 100 mg twice a day for 20 days in combination with hydroxychloroquine 400 mg daily for 3 months, following the Yoshinari protocol. A progressive improvement in headaches and joint pain was observed, and other symptoms did not appear in the following two years of follow-up.

### 3.4. Case 4

A 60-year-old Argentine woman who has lived in Italy for a year, and previously lived in Brazil from the age of 12 to the age of 30, reported a bite on her left forearm by a tick, identified as *Amblyomma cajennense* when she was 30 years old. A month later, she developed an erythema migrans around the bite and, subsequently, headache with photophobia and recurrent erythema nodosum. At that time, anti-Borrelia IgG antibodies by ELISA and PCR for Borrelia in DNA from peripheral blood were positive. The clinical picture met three major criteria for BYS.

In 2016, she presented in Italy with headache, low-grade fever, and myoarthralgias in her shoulders and knees, and hips. Tests for autoimmune diseases, anti-nuclear antibodies (ANAs), and anti-extractable nuclear antigens (ENAs) were negative. Anti-Borrelia IgG and IgM by CLIA resulted negative. Taking the previous diagnosis of BYS, the patient was treated with ceftriaxone, minocycline, and hydroxychloroquine, according to the Yoshinari protocol, with the improvement of the clinical manifestations.

### 3.5. Case 5

**Figure 3:**
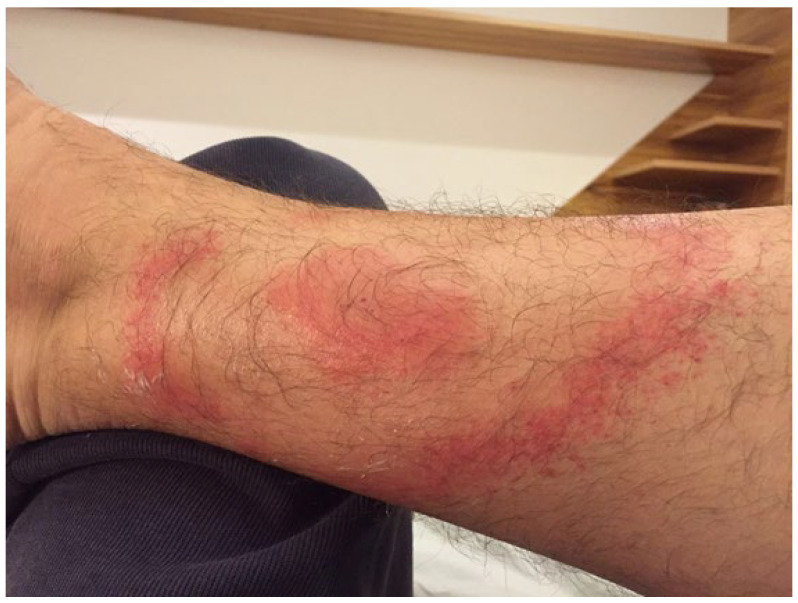
Bull’s eye erythema migrans, seen after 3 weeks from the tick bite in patient 5.

A 40-year-old Brazilian man living in São Paulo was bitten by a tick in the Amazon rainforest on the volar aspect of the distal third of the right forearm. Erythema migrans, with the typical “bull’s eye” appearance, developed after two weeks, as depicted in Figure 3. The patient also had fever and headache. Positive anti-Borrelia antibodies in an IgM ELISA were confirmed by Western blot (OspC); ANAs were negative. The patient was treated according to the Yoshinari protocol (ceftriaxone iv followed by doxycycline and hydroxychloroquine). EM and fever disappeared after one week and headache after two weeks.

## 4. Discussion

In this study, we report five patients diagnosed with BYS. All patients met the diagnostic criteria of BYS, including having been in risk areas in Brazil and having developed erythema migrans and/or other systemic manifestations [4]. In four patients, antibodies against *Bbls* were also detected, while in patient 2, only PCR supported the presence of Borrelia. In addition, patient 2 experienced relapsing symptoms and cognitive disturbances, representing minor diagnostic criteria for BYS [4]. Lyme disease in Europe is mostly characterized by the development of erythema migrans in 70–80% of patients and in BYS in 50% [12]. When this symptom is recognized, antibiotic treatment can be started with any further test for an early eradication of Borrelia. Also, Baggio–Yoshinari syndrome can manifest with erythema migrans; however, the clinical picture and treatment for this borreliosis differ from typical Lyme disease. While LD is usually treated with specific antibiotics, including amoxicillin, doxycycline, and ceftriaxone, BYS is treated with antibiotics and hydroxychloroquine for longer periods. The latter is used to modulate the immune-mediated inflammatory response, blocking the release of pro-inflammatory cytokines such as IL-6 [2] and treating persistent arthritis [13]. Clinically, BYS is distinguished from LD by its prolonged clinical evolution, high frequency of relapses, and the appearance of autoimmune manifestations [14], as shown even in our patients. In addition, immunoreactivity to *Bbsl* in BYS is usually limited in intensity and time. Differences in the clinical picture between LD and BYS rely on the development of erythema nodosum, observed in three patients described here. This dermatological manifestation is not usually observed in the classic form of LD, nor the high frequency of relapses [4]. Although non-specific, BYS patients often experience headaches, even in the early manifestation of the disease. Despite significant similarities between LD and BYS, the different clinical features can reflect the microbiological and ecological differences in BYS, which is vectored by *Amblyomma cajennense,* less frequently by *Rhipicephalus sanguineus,* and not by *Ixodes* ticks [15]. This could have forced Borrelia to adapt to survive and infect the Brazilian ticks [2]. Based on a previous study, the adaption of Borrelia to the different ecosystems determines the permanent preservation of the round shape instead of the conventional corkscrew morphology [16]. Borrelia can, indeed, undergo structural transformations, forming dense round bodies and outer membrane vesicles (OMVs; previously termed as blebs) when in unfavorable environments or conditions [17]. Pleomorphism, in general, may also help the Borrelia to evade the immune system or decrease antibiotic susceptibility, as well as change its pathogenic mechanisms [18], explaining the higher relapses and longer antibiotic treatments for BYS. In Borrelia, flagella not only provide the motility function but also confine the cell shape [18]. Flagella were visualized inside the round bodies of *Borrelia burgdorferi* [18]. In BYS, sequencing data limited to the Flagellin E gene showed a difference in 2–8 substitutions, discriminating BYS Borrelia [19] for partial characterization. Since Borreliae causing BYS have not been isolated by culture and fully sequenced, it is not known if a specific Borrelia genotype is the causative agent of BYS. At present, the evidence is that Borrelial DNA was detected in patients with BYS [9] and that Borreliae have atypical morphologies, as evidenced by focus floating microscopy [3]. Taking those observations, the diagnosis of BYS in comparison to classical LD can only be based on epidemiological and clinical data. A tick bite from *Amblyomma cajennense* in Amazonia and the onset of an erythema migrans are enough for the diagnosis of BYS. The infection caused by *Borrelia* permanently maintaining pleomorphic forms can justify the several peculiarities observed in BYS including the difficulty in cultivating the causative agent in the BSK medium, the absence of spirochaetes in the typical helical presentation, the low immunological response against *B. burgdorferi* in BYS patients, the frequent clinical relapses and the emergence of immune and allergic disorders. Nevertheless, we acknowledge that the etiology of BYS needs to be confirmed through a positive cultural examination or new technologies [20].

## 5. Conclusions

Clinicians must acknowledge that Baggio–Yoshinari syndrome exists in Brazil and Amazonia. Expanding the differential diagnosis for ill-returned travelers from Brazil may facilitate pre-travel guidance on tick-prevention measures and shape post-travel care in symptomatic patients choosing the proper therapeutic protocol. If not recognized and treated early, BYS can evolve with recurrent systemic complications, resulting in a chronic disease with neurological and joint symptoms that can be followed by autoimmune manifestations [21].

## Figures and Tables

**Table 1:** Summary of the cases.

CASES		Patient 1	Patient 2	Patient 3	Patient 4	Patient 5
**Major Criteria**		**3 Points**	**2 Points**	**3 Points**	**3 Points**	**3 Points**
1	Epidemiology: Tick bite in risk areas	Amazonia *Ambliomma cajennense*	Amazonia tick bites	Amazonia *Ambliomma cajennense*	Amazonia *Ambliomma cajennense*	Amazonia tick bite (not identified)
2	Erythema migrans	Yes	No	No	Yes	Yes
	Systemic manifestations	Neurological, articular manifestations	Neurological, articular manifestations, fever	Neurological, articular manifestations	Neurological, articular manifestations, fever	Neurological manifestations
3	Serology	Pos	Neg	Pos	Pos	Pos
**Minor Criteria**		**0 Points**	**2 Points**	**2 Points**	**1 Point**	**0 Points**
1	Relapsing symptoms	Neg	Yes	Yes	Neg	Neg
2	Chronic fatigue	Neg	Yes	Neg	Neg	Neg
	Cognitive disturbances	Neg	Yes	Neg	Neg	Neg
3	Identification Spirochaetae in blood ^1^	Neg	Neg	Pos	Pos	Not performed

^1^ By PCR or focus floating microscopy.

## Data Availability

The original contributions presented in the study are included in the article, further inquiries can be directed to the corresponding authors.
